# Synaptic pruning following NMDAR-dependent LTD preferentially affects isolated synapses

**DOI:** 10.1016/j.isci.2025.113093

**Published:** 2025-08-29

**Authors:** Côme Camus, Léa Leval, Viviana Villicana-Munoz, Sarka Jelinkova, Benjamin Compans, Frédéric Gambino, Etienne Herzog, Daniel Choquet, Eric Hosy

**Affiliations:** 1 Interdisciplinary Institute for Neuroscience, University of Bordeaux, CNRS UMR5297, 33000 Bordeaux, France; 2 Bordeaux Imaging Center, University of Bordeaux, CNRS UAR 3420, INSERM US04, Bordeaux, France

**Keywords:** Neuroscience, Molecular neuroscience, Cellular neuroscience

## Abstract

Long-term depression (LTD) is an activity-dependent decrease in synaptic strength. This state initiates either a re-potentiation or a loss, aka pruning, of the synapse within hours to days following its induction. However, the precise relationship between LTD and synaptic pruning remains elusive. Using various imaging techniques and electrophysiology on neuronal cell culture, we reveal a direct relationship between LTD and synaptic pruning. All synapses that exhibit NMDAR-dependent LTD are pruned unless counteracted by input-specific activity. Additionally, we identify the post-synaptic ion channels pivotal for sensing pre-synaptic activity. Finally, we find that neighboring synapses displaying coordinated activity act as mutual safeguards against pruning. These distinctive properties mean that LTD-dependent synaptic pruning has a major impact on synapses that do not contribute to responses involved in network activity.

## Highlights

• Decrease in AMPAR content at the synapse is not sufficient to trigger spine los
• NMDA-dependent LTD leads to synaptic pruning, which can be counteracted by activity
• Coordinated activity of neighboring spines protects synapses from LTD-dependent pruning
• LTD-dependent synaptic pruning allows for circuit refinement

## Graphical abstract

**Figure:**
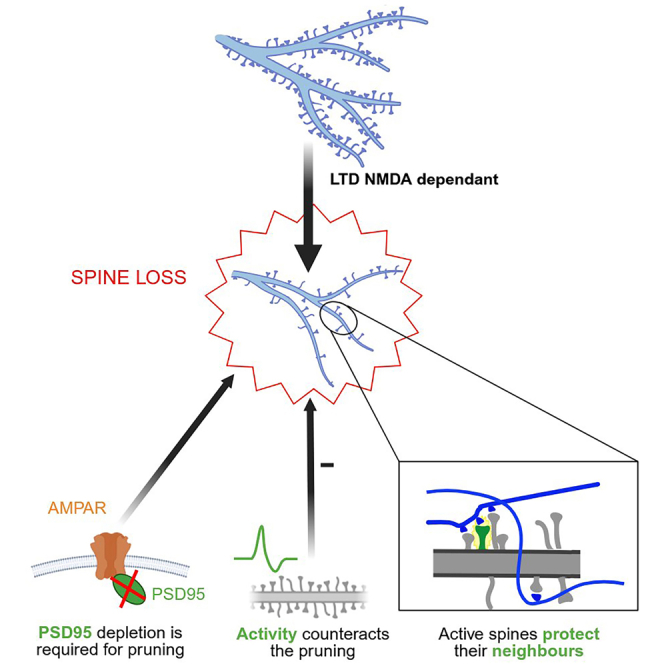

## Introduction

The number of synapses per neuron is not constant throughout its life. While synaptic plasticity modulates synaptic strength, structural plasticity shapes the size and the number of synaptic connections. This mechanism, which occurs during brain development and adulthood, does not impact all synapses equally but follows input specific rules.^1^ The creation or loss of synapses is crucial for refining brain connectivity and evolves concurrently with synaptic strength.^2^^,^^3^ During human brain development, activity-dependent synapse elimination reduces synaptic density by approximately 50%, resulting in the typical microarchitecture of the mature brain.^4^ Synaptic pruning, which is the loss of synapses, occurs throughout neuronal maturation^5^ and its dysfunction can lead to neurodevelopmental disorders such as autism spectrum disorder.^6^

Along with the developmental phase of spine selection, activity-driven changes in neuronal connectivity are essential for experience-dependent remodeling of brain circuitry, such as learning. *In vivo* studies have shown that learning is associated with pruning and that the level of spine loss is directly correlated with improved behavioral performance.^3^^,^^7^ However, as both synaptic and structural plasticity are interwoven mechanisms, it is difficult to determine their specific role during learning and memory.^8^ In the hippocampus, dendritic spine loss is driven through the induction of LTD, even if it occurs hours to days after it.^9^^,^^10^^,^^11^

Physiologically, LTD is a generic term mainly based on electrophysiological readouts and reflecting a global decrease in the evoked synaptic response when multiple pre-synaptic neurons are activated. This plasticity can be induced by various stimulation pathways, including glutamate-induced LTD through the activation of NMDAR or mGluR,^12^^,^^13^ insulin application,^14^ or activation of ATP by astrocytes following noradrenergic stimulation.^15^^,^^16^

Previous research has shown that the induction of NMDAR-dependent LTD can lead to synaptic pruning within hours to days post-induction, depending on the model and method of induction.^10^^,^^17^^,^^18^^,^^19^^,^^20^ However, it is unclear whether these two phenomena are mutually dependent or only share common signaling pathways.^8^^,^^21^ Furthermore, LTD induction at a spine does not always lead to its loss, as the LTD-pruning sequence often fails.^19^^,^^22^ Other studies have suggested that the initial state of synapses, including their activity, size, and network integration, can influence their fate.^10^^,^^19^^,^^23^ However, the specific conditions required for LTD to trigger synaptic pruning are still unclear. Moreover, it is unknown whether LTD is necessary for synaptic pruning or whether turnover of synapses can be induced independently of synaptic plasticity.

In this study, we aimed to determine which factors are necessary for the sequence of events that link NMDAR-dependent LTD and synaptic pruning. Using confocal imaging with electrophysiological recordings on live and fixed cultured hippocampal neurons, we first observed that the main molecular changes occurring during LTD induction must be intact to trigger pruning. We then identify several characteristics specific to synapses that become pruned following NMDAR-dependent LTD. Specifically, we found that pruned synapses had low to moderate activity previous activity and were often surrounded by a few weakly active neighboring synapses prior to their disappearance. Interestingly, the presence of multiple active pre-synaptic boutons from the same axon on a dendrite tended to protect synapses from pruning. These results suggest that neighboring synapses protect each other from pruning thanks to their concerted activity after LTD induction.

## Results

### NMDAR-dependent long-term depression triggers spine loss within 3 h

A 30-μM NMDA treatment for 3 min on Banker hippocampal cultured neurons triggered classical LTD, as evidenced by a subsequent decrease of approximately 30% in mEPSC amplitude within the following 30 min (mean amplitude ± SEM, 11.88 ± 0.57 vs. 8.4 ± 0.4, Figures S1A and S1B).

**Figure 1:**
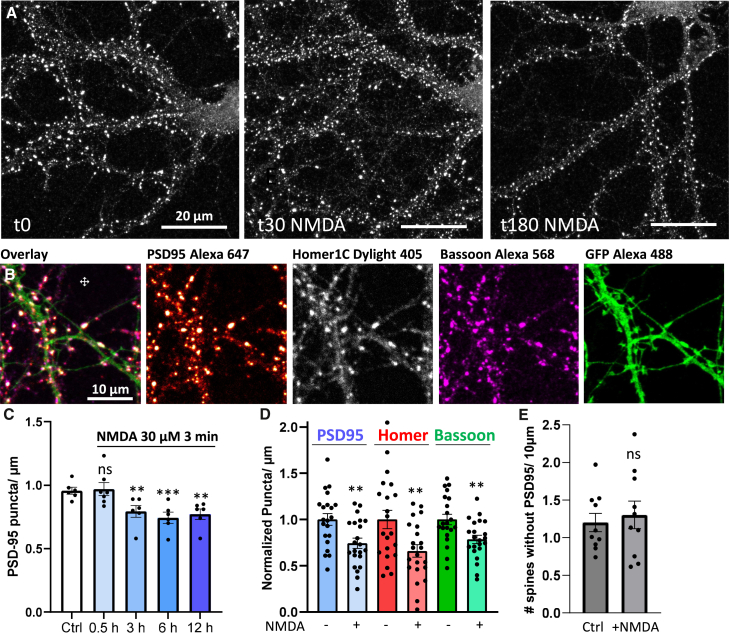
NMDAR-dependent LTD induces synaptic pruning (A) Example of confocal images after immunostaining of PSD-95. From left to right: Control, NMDA +30 min, NMDA +3 h. Scale bar: 20μm. (B) Example of 4-color confocal images, from left to right, an overlay, PSD95, Homer1C, Bassoon, and soluble GFP. Scale bar: 10μm. (C) Quantification of PSD-95 puncta density before, 30 min, 3, 6 and 12 h following LTD induction with NMDA. (D) Quantification of PSD-95, Homer and Bassoon puncta density before and 3 h after LTD induction with NMDA. (E) Quantification of spines presenting absence of PSD95 labeling before and 3 h after LTD induction with NMDA. Mean ± SEM, one dot represents one portion of dendrite. One-way ANOVA for C; t-test per labeling for D and E.

First, we investigated over time the effect of this treatment on PSD95 puncta density, a reporter of PSD. Thirty minutes after LTD induction, no modification of PSD95 puncta density was observed. However, significant decreases were measured at 3, 6, and 12 h post-induction (Figures 1A and 1C; mean ± SEM, 0.96 ± 0.03 without treatment, 0.97 ± 0.05 after 30 min, 0.79 ± 0.05 after 3 h, 0.74 ± 0.04 after 6 h, 0.77 ± 0.04 after 12 h).

To determine the extent to which various parts of the synapse are affected following NMDA treatment, we conducted four-color labeling of two PSD proteins (PSD95 and Homer1C), a presynaptic marker (Bassoon), and a volume marker (soluble GFP; Figure 1B). We then determined the degree of decrease in each labeling 3 h after NMDA-dependent LTD induction. Both the pre- and post-synaptic proteins decreased by a similar amount following the treatment, indicating that not only PSD95 was degraded but the entire synapse (including pre and post-synaptic components) was pruned (Figure 1D; mean density decrease ± SEM, 26 ± 6% for PSD95, 34 ± 7% for Homer1C, 23 ± 4% for Bassoon).

Additionally, we tested whether synapse pruning led to an increase in protrusions without PSD by quantifying the density of protrusions (filled with soluble GFP) lacking PSD95 labeling before and 3 h after LTD induction. No increase in protrusions without PSD was observed following NMDA treatment (Figure 1E, mean ± SEM, 1.2 ± 0.12 for control, 1.3 ± 0.18 after LTD). These experiments demonstrate that NMDA-dependent LTD triggers the elimination of both the post- and presynaptic organization, corresponding to synaptic pruning and the pruning of the entire spine.

To determine whether all types of post-synaptic LTD trigger similar synaptic pruning, we induced ATP-dependent LTD by activating P2X receptors. Three hours after induction, we determined whether PSD95 puncta density was affected.

One hundred μM ATP for 1 min induced a decrease of around 25% in mEPSC amplitude 30 min after the treatment. This was similar to the decrease observed after NMDA treatment (mean amplitude ± SEM, 11.88 ± 0.57 vs. 8.4 ± 0.4 and vs. 9.23 ± 0.52 after NMDA and ATP treatment, respectively: Figures S1A–S1C).

While NMDA induced a significant decrease (around 30%) in PSD95 puncta density after 3 h, no modification in spine density could be observed 30 and 180 min after ATP treatment (Figures S1D and S1E; mean PSD-95 puncta density ± SEM, for NMDA treatment: 0.8806 ± 0.026 at t0, 0.8626 ± 0.016 at t30, 0.6021 ± 0.015 at t180, Figure 1E; and for ATP treatment: 0.9136 ± 0.022 at t0, 0.9211 ± 0.022 at t30, 0.9367 ± 0.02 at t180). Therefore, the activation of NMDAR, but not P2XR, induced synaptic pruning, while the activation of both receptors triggered a similar decrease in synaptic currents.

### Synaptic pruning requires postsynaptic density 95 removal from synapses

In a previous study, we demonstrated that the maintenance of NMDAR-dependent LTD requires the phosphorylation of PSD-95 at the T19 position by GSK3β, which targets PSD-95 to autophagosomes for degradation.^16^ Here, we investigated whether the same pathway is required and necessary for the induction of NMDAR-dependent synaptic pruning.

**Figure 2:**
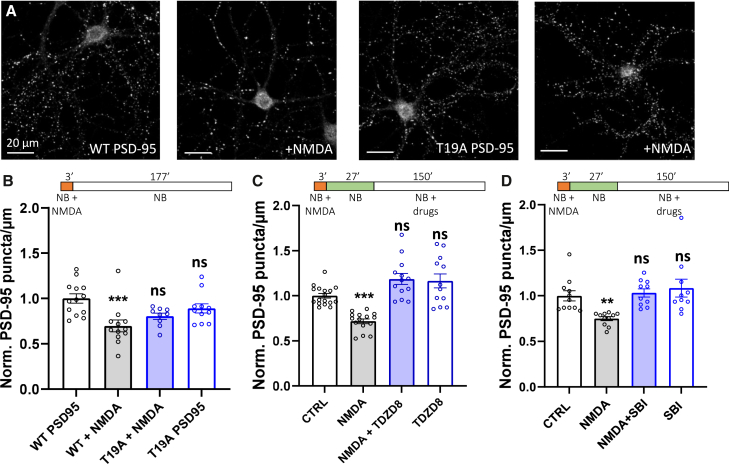
PSD-95 synaptic depletion is required for pruning (A) Example of confocal images after PSD-95 immunostaining. From left to right: WT PSD-95 overexpression, WT PSD-95 overexpression and NMDA +3 h, T19A PSD-95 overexpression, T19A overexpression and NMDA +3 h. (B, C and D) Quantification of PSD-95 puncta density before and 3 h after treatments, one dot represents the mean of a cell. (B) From left to right: WT PSD-95 overexpression, WT PSD-95 overexpression and NMDA +3 h, T19A PSD-95 overexpression, T19A overexpression and NMDA +3 h. Mean ± SEM, one-way ANOVA, for WT + NMDA, Dunnett’s multiple comparisons test gives *p* = 0.0008. (C) From left to right: control, NMDA, NMDA + TDZD8 30 min later, TDZD8. TDZD8 being an antagonist of GSK3β. Normalized data to control, mean ± SEM, for NMDA Tukey’s multiple comparisons test gives *p* = 0.0001. (D) From left to right: control, NMDA, NMDA and SBI-0206965 30 min later, SBI-0206965 alone. SBI-0206965 being an antagonist of autophagy. Mean ± SEM, one-way ANOVA, for NMDA, Dunnett’s multiple comparisons test gives *p* = 0.01.

First, we examined the effect on the LTD-dependent synaptic pruning of the expression of the PSD95 T19A mutant, which is unable to be phosphorylated by GSK3β (Figures 2A and 2B).^16^^,^^24^ The expression of the mutant had no significant effect on synaptic density before pruning induction (Figure 2B) but abolished the decrease in PSD-95 puncta 3 h after NMDA application, indicating the absence of synaptic pruning (normalized mean of PSD-95 puncta density ± SEM, for WT with NMDA: 0.69 ± 0.06; for PSD95 T19A alone: 0.89 ± 0.05 and with NMDA: 0.80 ± 0.03).

Then, we blocked either the GSK3beta activity or the autophagy processes 30 min after LTD induction and determined the synaptic density 2.5 h later (Figures 2C and 2D). We found that the application of the GSK3β inhibitor TDZD8 (10 μM) or the autophagy inhibitor SBI-0206965 (0.5 μM) for 2.5 h following LTD induction suppressed the decrease in PSD-95 puncta density, and thus synaptic pruning (normalized mean of PSD-95 puncta density ± SEM, for NMDA + TDZD8: 1.186 ± 0.06 vs. NMDA alone: 0.7179 ± 0.027; for NMDA + SBI: 1.031 ± 0.045 vs. NMDA alone: 0.7492 ± 0.022).

Taken together, these results indicate that (a) the induction of NMDAR-dependent LTD highly promotes the induction of spine pruning and that (b) the LTD pathway needs to remain active after LTD induction for pruning to occur.

### Post-long-term depression synaptic activity regulates synaptic pruning rate

**Figure 3:**
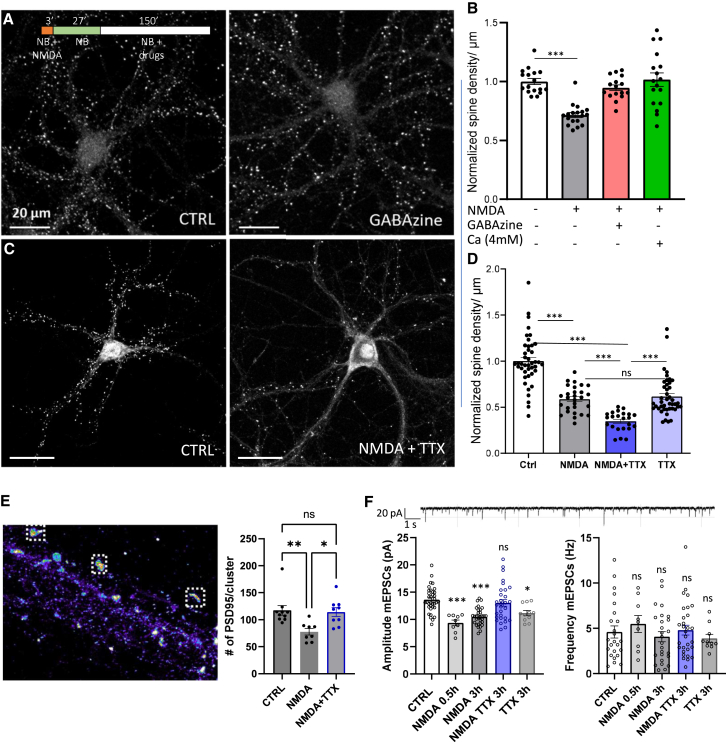
Network activity regulates pruning efficiency following NMDA-dependent LTD (A) Example of confocal images after PSD-95 immunostaining, left: control, right: NMDA and GABAzine +3 h. Scale bar: 20μm. (B) Quantification of PSD-95 puncta density, normalized data to control, gray: NMDA + 3h, blue: NMDA + GABAzine + 3h, green: NMDA+ 3h in 4 mM Ca^2+^. Comparison between NMDA and GABAzine and 4 mM Ca^2+^, Dunnett’s multiple comparisons test gives *p* < 0.0001. (C) Example of confocal images after PSD-95 immunostaining, left: Control, right: NMDA and TTX +3 h. Scale bar: 20μm. (D) Quantification of PSD-95 puncta density, White: Control, gray: NMDA + 3h, light purple: TTX + 3h, full purple: NMDA+TTX+3h. (E) (left) Example of d-STORM image of PSD95 nanoscale organization and (right) quantification of number of PSD95 molecules per spine before and after NMDA or NMDA + TTX treatment for 3 h. (F) AMPAR-miniature current amplitude (left) and frequency (right) with similar conditions to D. For B, D and F, each dot represents the mean of a cell, mean ± SEM, one-way ANOVA, and Dunnett’s posttest.

After 3 h of NMDAR-dependent LTD induction, around 30% of synapses are eliminated (Figure S1D). This suggests that not all depressed synapses behave similarly. Previous studies have shown a direct correlation between the level of synaptic activity and the lifespan of synapses.^10^^,^^19^^,^^23^ To investigate the effect of neuronal activity on LTD-induced spine loss, we increased neuronal activity 30 min after NMDAR-dependent LTD induction by inhibiting inhibitory neurons with a GABAA inhibitor (Gabazine 2 μM) or by increasing the probability of glutamate release by raising the extracellular calcium concentration to 4 mM (Figures 3A and 3B). Neurons were incubated in these conditions for 2.5 h, then synapse density was measured. Both conditions resulted in the complete abolition of synaptic pruning 3 h after NMDA application (normalized mean of PSD-95 puncta density ± SEM, for NMDA: 0.7159 ± 0.021, for NMDA + Gabazine: 0.9473 ± 0.024, for NMDA + 4 mM calcium: 1.016 ± 0.057; Figures 3A and 3B). This indicates that elevated synaptic activity counters the LTD-dependent synaptic pruning process.

Next, we investigated the impact of decreased network activity on synaptic pruning (Figures 3C and 3D). Thirty minutes after NMDAR-dependent LTD induction, we incubated the neurons with TTX (0.5 μM) for 2.5 h and then measured synaptic density (normalized mean of PSD-95 puncta density ± SEM, for control: 1 ± 0.05, for NMDA: 0.59 ± 0.03, for TTX: 0.51 ± 0.02; for NMDA + TTX: 0.34 ± 0.02). More than 65% of all synapses were eliminated within 3 h of LTD induction when action potentials were blocked in the network.

We then combined super-resolution imaging technique and electrophysiology to describe the properties of the remaining synapses as compared to control naive ones (Figures 3E and 3F). We determined by d-STORM the amount of PSD-95 per PSD before and after NMDA treatment for 3 h in the presence or the absence of TTX. No significant difference in PSD-95 quantity per PSD was observed in the presence of TTX, while NMDAR-dependent LTD triggers a significant decrease in PSD-95 content in the absence of TTX.^16^ This suggests that the synapses remaining after NMDA treatment in the presence of TTX did not undergo proper LTD. To confirm this, we measured miniature current amplitudes and frequency (Figure 3F). Thirty minutes and 3 h after NMDA treatment, a 30% decrease in synaptic miniature currents was measured. However, when TTX was applied 2.5 h after LTD induction, no decrease in synaptic currents was observed. These results collectively indicate that the synapses remaining after NMDA and TTX treatment did not undergo LTD. In parallel, the miniature frequency did not decrease (Figure 3F right panel) while 66% of synapses were pruned (Figure 3D) following NMDA and TTX treatment. First, this indicates that a high percentage of synapses exhibiting LTD are removed in the absence of network activity, while the remaining ones do not show depressed currents. Second, either primarily synapses with a low miniEPSC frequency are pruned while the more presynaptically active ones are preserved, or, under our conditions, a form of presynaptic plasticity occurs that increases miniEPSC frequency at the remaining synapses.

### Activation of AMPAR and L-type calcium channels is related to the maintenance of synapses

**Figure 4:**
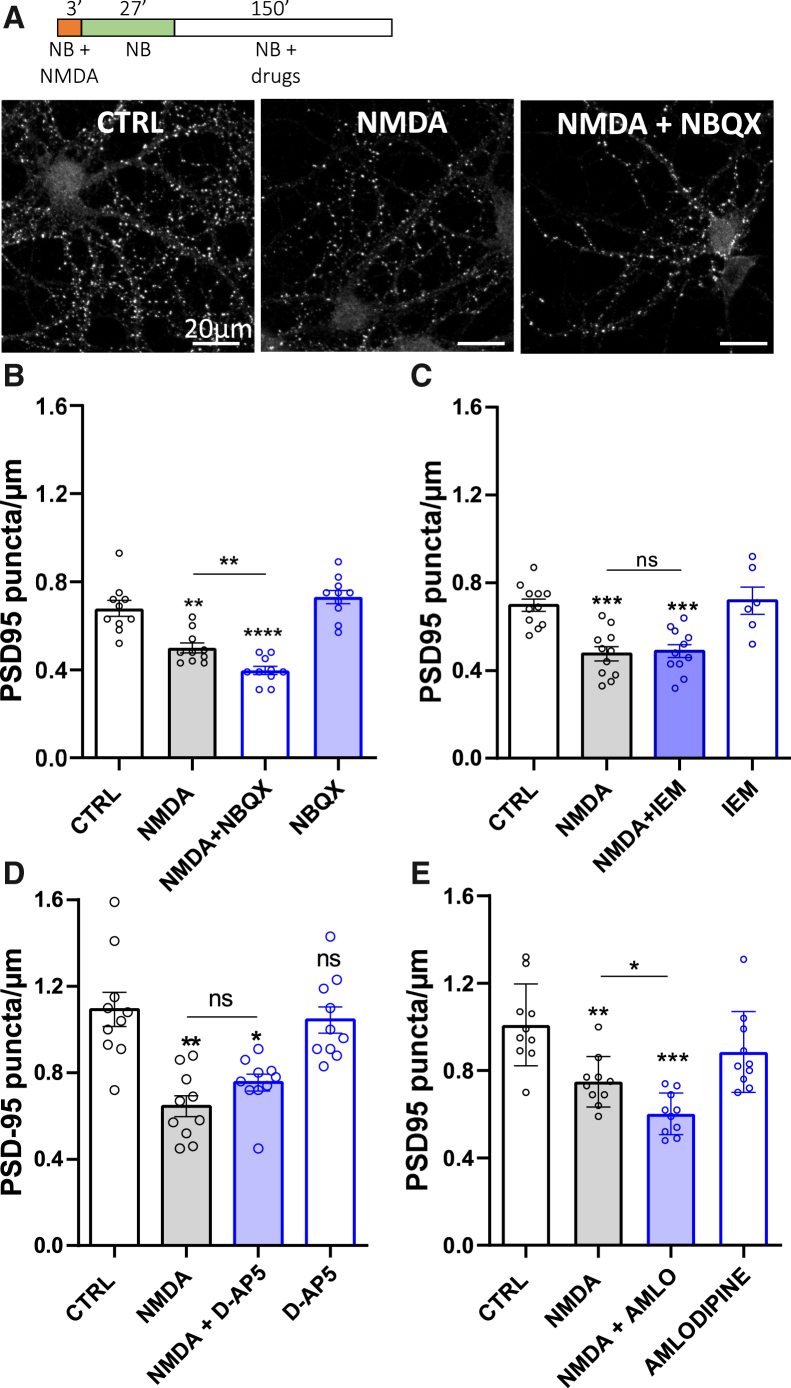
Blockade of AMPAR and L-type calcium channels increases pruning (A) Representative confocal images of PSD-95 immunolabeling after NMDA treatment in the presence or absence of NBQX. (B, C, D, and E) Quantification of PSD-95 puncta density 3 h after the beginning of treatments: one dot represents the mean of a cell. Conditions are detailed below the histograms. (B) Represents the effect of NBQX, a global inhibitor of AMPAR. (C) Represents the effect of calcium-permeant AMPAR that are blocked with IEM1460. (D) Represents the effect of D-AP5, a specific inhibitor of NMDA receptors. (E) Represents the effect of amlodipine an inhibitor of L-type voltage dependent calcium channels. Mean ± SEM, one-way ANOVA, Tukey’s multiple comparisons post-tests.

We investigated the molecular mechanism responsible for spine maintenance/loss. The pruning efficiency was evaluated 3 h after LTD induction by blocking various ion channels responsible for synapse depolarization or calcium entry (Figure 4). The protocol consisted in inducing complete LTD by NMDA treatment and 30-min incubation, followed by the application of inhibitors of AMPAR receptors (NBQX, 10 μM; Figure 4B), calcium-permeable AMPAR (IEM 1460, 100 μM; Figure 4C), NMDAR (D-AP5, 50 μM; Figure 4D), and an L-type voltage-dependent calcium channel (Amlodipine, 5 μM; Figure 4E) for 2.5 h. The application of the AMPAR antagonist 30 min after NMDA treatment significantly increased pruning at 3 h (Figure 4B), meaning that AMPAR are important to counteract synaptic pruning after LTD. Conversely, the application of the Ca^2+^-permeable AMPAR antagonist or NMDAR antagonist did not affect pruning rate (Figures 4C and 4D). This suggests that calcium entry, which is probably responsible for protecting synapses from pruning, is not mediated by NMDAR or calcium-permeable AMPAR, revealed by APV and IEM treatment. The application of the L-type voltage-dependent calcium channel inhibitor 30 min after NMDA significantly increased pruning intensity (mean density of PSD95 puncta ± SEM, for NMDA alone: 0.749 ± 0.004; for NMDA + amlodipine: 0.602 ± 0.03) (Figure 4E). These results indicate that the maintenance signal that counteracts LTD-dependent synaptic pruning is mediated by AMPAR-induced depolarization, leading to the activation of L-type calcium channels that enable calcium entry.

### Isolated synapses are preferentially pruned

**Figure 5:**
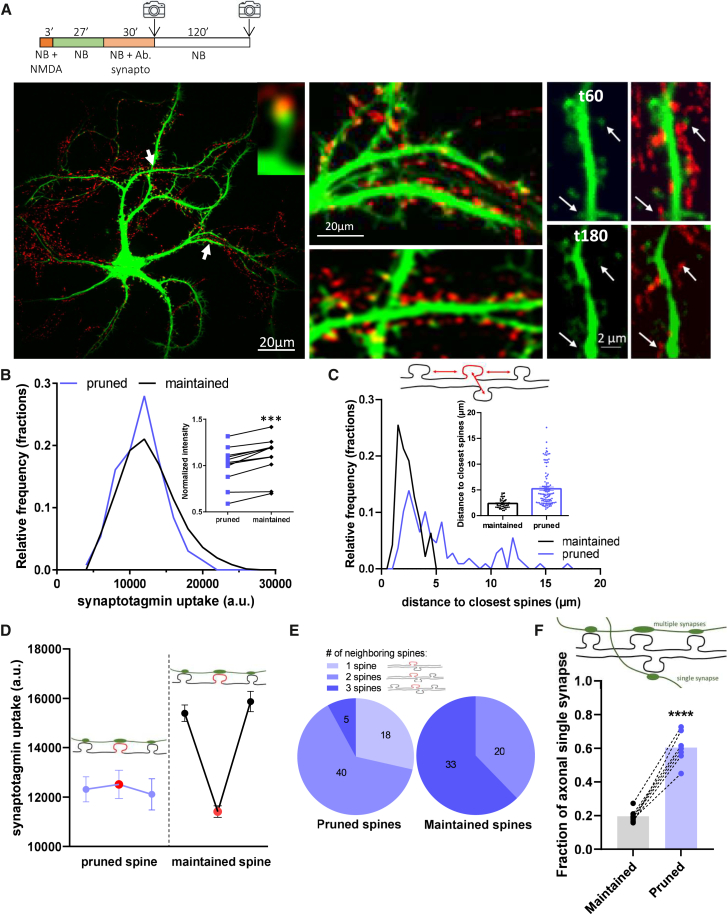
Active synapses protect their neighbor from pruning (A) Representative image of confocal image of a GFP-transfected neuron (green) combined with synaptotagmin-1 labeling by uptake experiments (red). Left: arrows indicate portions of dendrites zoomed in the middle panels. Right: images of the same portion of dendrite, on the left for the EGFP signal alone and on the right merged with the anti-synaptotagmin signal. At the top, 30 min after LTD induction with NMDA, and 3 h after at the bottom. Arrows indicate synapses pruned at 3 h (bottom). (B) Frequency distribution of initial synaptotagmin uptake for maintained spines at 3h (black) and pruned spines at 3h (blue). Inset shows the synaptotagmin uptake per neuron at the maintained and pruned spines. t-test, *p* < 0.0001. (C) Distribution of the distance between spines and their closest neighbors, for maintained spines at 3h (black) and pruned spines (blue). Maintained spines were selected to present a similar level of activity as that of pruned ones (mean spines ±2000 au). Inset is the histogram of these distances, Mean ± SEM, t-test gives *p* < 0.0001. (D) Synaptotagmin uptake intensity of surrounding spines (at less than 5 μm) maintained spines (black) or pruned spines 3 h after NMDAR-dependent LTD induction. Labeling intensity of the spine of interest is represented by the red spot: neighboring spines are on the left and right (purple or dark). Mean ± SEM, Tukey’s multiple comparisons test gives *p* < 0.0001 for neighbors of pruned spines, and *p* = 0.0004 for comparison between neighbors of pruned and maintained spines. (E) Number of spines in the 5 μm vicinity of spines, constituting clusters of spines for maintained and pruned spines. (F) Fraction of synapses connected by “en passant” bouton for the maintained and pruned spines. Dashed lines connect groups of spines from the same neuron, t-test, *p* < 0.0001.

To determine the activity-related rules responsible for synaptic fate following NMDAR-dependent LTD, we correlated pruning with pre-synaptic synaptotagmin-1 uptake, which reflects pre-synaptic activity.^25^ LTD was induced by NMDA treatment and maintained for 30 min in culture medium to be fully implemented. Then, pre-synapses were loaded with synaptotagmin antibody coupled to a pH-sensitive fluorescent probe (CypHer 5E) for 30 min, and an image of post-synapse (GFP) and synaptotagmin labeling (CypHer) was taken. Two hours later, another GFP image was taken to determine which synapses had been pruned (Figure 5A).

Although variable, the synaptotagmin labeling intensity of maintained synapses per neuron was always higher than that of pruned ones (Figure 5B insert, normalized mean for maintained: 1.096 ± 0.069). In addition, the maintained synapse population presented an enrichment toward higher synaptotagmin intensity (Figure 5B). We then determined the distance between the pruned or maintained synapses and their closest neighbors (Figure 5C). Pruned synapses had a broader distribution of distance from their neighbor than the maintained ones. 89% of maintained synapses had a neighbor closer than 4 μm before pruning, while only 46% of pruned synapses had a neighbor within the 4 μm, suggesting that the neighboring synapses in μm range exert a protective effect against pruning after LTD.

To determine whether the activity of neighboring synapses plays a role in protecting spines from pruning, we investigated the activity level of neighboring synapses and its impact on synaptic fate (Figure 5D). Synapses surrounding the maintained synapses (dark dots) had a 30% higher level of activity than those surrounding the pruned ones (blue dots) (mean ± SEM, for maintained synapses: 15,633 ± 264.8; for pruned synapses: 12,213 ± 402). This means that for pruned and maintained synapses with a similar level of activity and having neighboring spines within 5 μm, the activity of the latter plays a role in their fate. This reinforces the notion of a protective effect exerted by neighboring synapses against pruning. To avoid a synapse from being pruned, not only is its own activity decisive in its fate, the presence of surrounding active synapses seems also important.

To complete the description of the synaptic environment that determines spine maintenance or loss, we counted the number of neighbors within the 20 μm around the pruned or maintained synapses (Figure 5E). The pruned synapses were more isolated than the maintained ones. 29% of pruned spines were surrounded by only one synapse, 63% by two, and 8% by three, whereas the maintained ones were comprised of groups with two (37%) or three (63%) other synapses, and none with only one. Altogether, these results indicate that synapses tend to protect each other from pruning thanks to their proximity.

Finally, we determined whether belonging to a cluster of spines receiving input from the same axon improves protection from pruning for a synapse (Figure 5F). To study the number of synapses that axons make with dendrites, we used synaptotagmin labeling to estimate the number of pre-synaptic boutons per axon on a dedicated dendrite. We observed that an isolated synapse of a dedicated axon was the most likely to be pruned, while neighboring post-synapses connected to the same axon were mainly maintained after LTD treatment. 60% of pruned synapses were connected to an “en-passant” bouton, while only 20% of the maintained ones were.

### Network activity is weakly affected by long-term depression-dependent synaptic pruning

Various patterns of neuronal activity are visible in neuronal networks without external stimulation. These include miniature currents corresponding to a single synapse response, coordinated poly-synaptic responses where multiple synapses from the same axon respond together, and sequential poly-synaptic responses where the recorded neuron receives multiple stimuli from various axons throughout the burst duration (Figure S2). It has been suggested that weakly integrated synapses, receiving few coordinated inputs, are pruned following LTD.^10^ This suggests that polysynaptic responses should be less affected by LTD-dependent pruning than individual responses.

**Figure 6:**
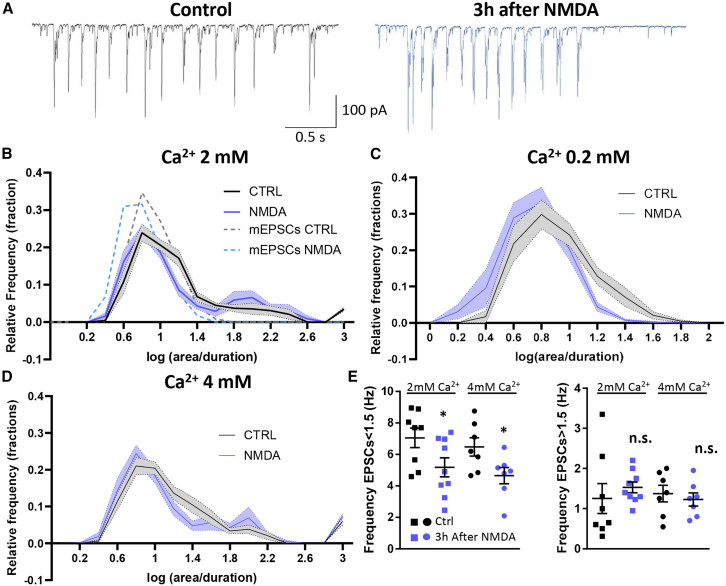
Pruning does not affect poly-synaptic responses (A) Representative traces of spontaneous EPSCs recordings on neuronal cell culture. Before (black) and 3 h after NMDA-dependent LTD induction (blue). (B, C, and D) Frequency distribution of log(area/duration of event) of spontaneous EPSCS recorded with 2 mM, 0.2 mM, and 4 mM of extracellular Ca^2+^, respectively. Dashed lines in B represent the mEPSC distribution measured in Figure 1A. (E) Frequency of currents presenting log(area/duration of event) value < 1.5 (left) and >1.5 (right) before (dark) and 3 h after (blue) LTD induction, with 2 or 4 mM of extracellular Ca^2+^. Mean ± SEM, unpaired t-test.

To investigate this issue, spontaneous EPSCs were measured in control conditions and 3 h after NMDA application (Figure 6A) at three different extracellular calcium concentrations (0.2, 2, and 4 mM) to vary the probability of release. Two populations of EPSC amplitude were identified at 2 and 4 mM of extracellular calcium in the recording chamber (Figures 6B and 6E). The first population, with a log (area/duration) < 1.5, corresponded to single synapse responses and presented a shift toward smaller areas after NMDA treatment that was related to the decrease in AMPAR content induced by LTD. This population, which represented the only current type observed at 0.2 mM of calcium, exhibited a 10.6% decrease in area (mean log(-area/duration) +/− SEM from 0.9249 ± 0.009 to 0.8271 ± 0.008, 3 h after LTD induction).

The second population of synaptic responses with a log(area/duration) > 1.5 corresponded to poly-synaptic responses. The larger area of these more complex responses is mainly due to the number of recruited synapses instead of mono-synaptic responses. After LTD, their average intensity was not affected, indicating that LTD-dependent pruning tends to maintain the number of synapses bursting together.

Comparison of mono and poly-synaptic response frequency revealed a significant decrease in mono-synaptic responses 3 h after NMDA treatment in both 2 and 4 mM of calcium (t-test: *p* = 0.048 and 0.037), while there was no decrease in poly-synaptic frequency (Figure 6E).

This set of experiments indicated that LTD-dependent synaptic pruning, which suppresses 35% of total synapses, mainly affects synapses that do not contribute to responses involved in network (poly-synaptic) activity.

## Discussion

The findings of this study are 2-fold. First, NMDAR-dependent LTD directly triggers synaptic pruning. All synapses that express LTD following NMDAR activation are lost within a few hours unless some activity counteracts the induction of pruning. Second, the rules governing pruning in cell cultured model favor the maintenance of network-integrated synapses to the detriment of the isolated and/or inactive synapses, which tend to be removed.

### Postsynaptic density-95 reshuffling mediates the relationship between long-term depression and synaptic pruning

NMDA treatment of neuronal cell cultures induces chemical LTD, resulting in a 35% decrease in synaptic density within 3 h. This decrease affects the pre-synapse (bassoon), the PSD (PSD95 and Homer1C) and the spine to a comparable degree and leads to the complete loss of both molecular components of the synapse and of the protrusion forming the spine. The entire study was conducted on neuronal cell cultures at DIV 14. It is conceivable that the young age of the neurons could result in mechanisms that are lost at later developmental stages or in other models, such as brain slices or *in vivo*.

The relationship between the number of AMPARs per PSD and synapse selection has been extensively investigated. One prevailing view is that a decrease in AMPAR synaptic content is both necessary and sufficient to initiate synaptic pruning.^26^ To test this hypothesis, we used P2XR activation to induce LTD, which produces a comparable reduction in synaptic current without significantly altering the organization of PSD. Specifically, P2XR treatment only leads to an increase in AMPAR internalization.^15^^,^^27^ Our findings demonstrate that, unlike NMDAR-dependent LTD, P2XR-dependent LTD does not induce synaptic pruning within the 3-h time frame (as depicted in Figure S1). This implies that the decrease in AMPAR current is not the cause of synaptic pruning but rather a consequence of LTD induction. The disparity in pruning between the two protocols might be due to the signaling cascade that triggers the removal of PSD-95 from synapses, which has previously been identified as a critical marker of LTD-induced synaptic pruning.^28^^,^^29^

Since the effective reduction in AMPAR synaptic currents is not the determining factor of pruning induction, we investigated the effects of a 30-min induction of LTD followed by the inhibition of the late phase of LTD. This phase corresponds to the removal of PSD95 and its targeting to the autophagic pathway, potentially initiating spine selection (as shown in Figure 2). Our data show that pruning is prevented by blocking the reshuffling of PSD95 through the expression of the T19A mutant or inhibiting the removal of PSD-95 30 min after LTD induction with GSK3β or autophagy inhibitors.

Molecularly, LTD induces PSD-95 depalmitoylation, detachment from α-actinin, and phosphorylation by GSK3β, all occurring within the first 20 amino acids of PSD-95.^30^ This signaling cascade increases the pool of mobile PSD-95, which is crucial for initiating spine loss.

The experiments highlight the direct relationship between LTD and synaptic pruning. It is even conceivable that the role of LTD is to initiate pruning, thereby placing the synapse in a state of anticipation to determine whether it should be maintained or pruned.

This hypothesis is further supported by experiments shown in Figures 3C–3E. When network activity was suppressed by TTX 2.5 h after a 30-min NMDAR-dependent LTD induction, nearly 70% of the PSD95 puncta had disappeared. Interestingly, the remaining 30% did not exhibit signs of LTD, either in terms of nanoscale organization and PSD95 content or regarding AMPAR currents, which were similar to the control. This suggests that potentially all spines that exhibit LTD are pruned in the absence of network activity. One interpretation could be that NMDAR activation-induced LTD serves as the initial step of synaptic pruning, which can be counteracted by synaptic activity.

### Rules to block the long-term depression-pruning relationship

Prior studies have suggested that the induction of LTD does not consistently result in spine loss,^10^^,^^19^^,^^23^^,^^31^ with evidence indicating the potential recovery of synapses from depressive states. In agreement with these findings, our results show that during typical network activity, NMDAR-dependent LTD leads to the removal of 30%–40% of spines, whereas 70% vanish in the absence of network activity (refer to Figures 1 and 3) and 47% when the AMPAR response is blocked (Figure 4A). This suggests that the LTD-pruning sequence is disrupted in 30%–40% of cases.

We further explored factors influencing synaptic maintenance post-LTD induction by manipulating network activity. By elevating extracellular calcium concentrations and inhibiting inhibitory neuron activity, we were able to prevent synaptic pruning completely, thereby underscoring the capacity of high network activity to counteract LTD-dependent pruning.

Regarding the activity dependence of synaptic pruning, a small subset of highly active synapses was resistant to loss, while individual activity did not consistently determine the fate of the majority of synapses.^23^^,^^32^ The proximity and activity of neighboring synapses appeared to be crucial. The synapses that were maintained had closer neighbors and higher activity levels (Figure 5), suggesting a form of collaboration between clustered synapses to avoid removal.

The protective effect of nearby active synapses is likely linked to local calcium influx. GABAergic inhibition is known to limit the diffusion of calcium into dendritic branches.^33^ Therefore, local calcium influx near active spines may foster the maintenance of the surrounding synapses. Alternatively, calcium could activate various molecular pathways, thereby activating a calcium-sensitive protein and allowing its subsequent diffusion along the dendritic branch as a maintenance signal.^34^

Nevertheless, these findings seemingly conflict with those of previous studies. For example, LTP induction at the surrounding synapses was found to trigger the synaptic pruning of the non-potentiated synapse within the group.^23^ Developmental studies suggest that active spine proximity promotes the removal of low-active spines rather than their maintenance.^35^ However, heterosynaptic shrinkage, pruning, and developmental-specific synaptic selection involve distinct molecular reshufflings and pathways compared to homosynaptic pruning.^8^ This difference may explain the variable influence that neighboring spines exert on each other, highlighting the intriguing variability in rules governing synaptic interaction and recovery signals in the different paradigms of synaptic plasticity and selection.

### Activation of VGCCs constitutes a maintenance signal for synapses

When seeking the molecular source of calcium responsible for spine maintenance, our initial focus was on the NMDAR. Indeed, NMDA receptor activity is a major activity-dependent contributor to calcium entry at spines. However, inhibiting NMDA 30 min after LTD induction and throughout the entire pruning experiment did not influence the number of pruned and maintained synapses. For this reason, we turned our attention to the AMPAR side and discovered that the process is AMPAR-dependent but is not mediated by calcium-permeant AMPAR.

Ultimately, calcium entry stems from the activation of L-type voltage-dependent calcium channels induced by AMPAR-triggered depolarization. The inter-dependence between AMPAR and VDCC has already been described in Thiagarajan et al., where 24-h inhibition of AMPAR leads to a similar effect as L-type channel inhibition on synaptic strength increase.^36^ This reflects that AMPAR-dependent synaptic depolarization is enough to activate VDCC, triggering various transduction cascades, either spine maintenance or homeostatic plasticity. Moreover, these findings align with the concept of local dendritic depolarization and synaptic integration. When spines cooperate and especially when they are activated simultaneously because they belong to the same axon, a local summation of depolarization occurs that promotes the activation of voltage-gated calcium channels present both at the spine and on the dendrite.

### Circuit refinement and the theory of noise reduction

In addition to unraveling the mechanisms of pruning, we explored the consequences of synaptic pruning on neuronal integration. Our findings on spontaneous synaptic activity indicate that pruning tends to enhance the segregation of poly-synaptic response (Figure 4). Unlike poly-synaptic signaling, mono-synaptic current population diminishes after pruning, thereby reflecting the preference for pruning to impact “axon to dendrite” single-connexion (Figure 5F). Consequently, the total number of “en-passant” synapses decreases compared to the number of multiple-connected axon-dendrite population (Figure 5I).

Considering the refinement of inputs resulting from pruning, one must contemplate the notions of synchrony and aberrance in synaptic signaling for neuronal integration. Pruning selectively affects isolated synapses spatially and in terms of activity, reducing the number of spatially isolated synapses with few neighbors and synapses firing alone. This suggests that pruning refines the network by eliminating aberrant signaling, akin to noise, compared to poly-synaptic coordinated signaling. Consequently, synaptic pruning likely enhances the efficacy and specificity of information transmission in a neuronal network, as already observed *in vivo*.^37^

In conclusion, we established that LTD-related pruning is not associated with a decrease in synaptic currents but with NMDAR-induced removal of PSD-95 from synapses. Despite this, we observed synapse recovery from LTD induction through the activation of L-type calcium channels. Notably, the maintenance pathway involves cooperation between highly active and low-active synapses, specifically impacting spatially and activity-isolated synapses. The molecular basis of such cooperation is still undefined. This could be related to HRAS diffusion, as described following LTP, or to any other calcium-dependent pathway.^38^ These overall results represent a novel insight strongly suggesting the implication of synaptic pruning in promoting coordinated synaptic inputs.

### Limitations of the study

This study has limitations. First, the neuronal cell culture model exhibits differences in development and activity compared to *in vivo* conditions, which may limit the direct translation of our findings. For instance, homeostatic plasticity does not occur under our culture conditions, which is a noteworthy distinction.

Another important limitation concerns neuronal connectivity. In the brain, a neuron typically receives a single or a few inputs of a given axon, whereas cultured neurons are not subject to such constraints. As a result, some of our experimental outcomes may be influenced more by the coordinated firing of presynaptic neurons from the same region—as described by Takahashi et al.^37^ —than by multiple synaptic connections from a single axon.

## Resource availability

### Lead contact

Further information and requests for resources should be directed to and will be fulfilled by the lead contact, Hosy Eric (eric.hosy@u-bordeaux.fr).

### Materials availability

No new materials (i.e., reagents) were created in the process of this study. Plasmids are available under request.

### Data and code availability

• All data of the article are available upon request.
• No code has been developed for this study.

## Acknowledgments

We acknowledge the Bordeaux Imaging Center, part of the FranceBioImaging national infrastructure (ANR-10INBS-04-0, for support in microscopy). We thank the IINS cell biology core facilities (LABEX BRAIN [ANR-10-LABX-43]) and in particular C. Breillat, E. Verdier, and N. Retailleau for cell culture and plasmid production. This work was supported by funding from the Ministère de l’Enseignement Supérieur et de la Recherche (ANR SyTune ANR-21-CE37-0010 and SytGAP ANR-21-NEUC-0003 and E.U. Horizon-Hlth-2022-Disease-06 (101080580) to E.H.) 10.13039/501100004794Centre National de la Recherche Scientifique (CNRS), 10.13039/501100000781ERC grant DynSynMem (787340) to D.C., 10.13039/501100002915Fondation pour la Recherche Médicale fellowship to B.C. 10.13039/501100000780EU funding HORIZON-MSCA-2021 EaSYFUN–10106258 to S.J. & Et.H. and ANR-20-COEN-0003-02 LocalDementia to Et.H. S.J. Funded by the 10.13039/501100000780European Union. Views and opinions expressed are those of the authors only and do not necessarily reflect those of the European Union or the 10.13039/100020668European Research Executive Agency (REA). Neither the 10.13039/501100000780European Union nor the granting authority can be held responsible for them. Portions of the article were developed from the thesis of C.C.

## Author contributions

C.C. and L.L. performed all dSTORM, electrophysiology, and synapse counting. E.H conceived and supervised the study. Et.H. and S.J. developed some techniques. E.H. financed the study. F.G. helps to conceptualize some experiments. C.C., E.H., and D.C wrote the article. All authors contributed to the revision of the article.

## Declaration of interests

The authors declare no competing interests.

## STAR★Methods

### Key resources table

**Table:** 

REAGENT or RESOURCE	SOURCE	IDENTIFIER
**Antibodies**		

monoclonal mouse anti-PSD-95 antibody	ThermoFischer	RRID: AB_2092361
monoclonal rabbit anti-Bassoon	Synaptic Systems	RRID: AB_887697
monoclonal Guinea Pig anti-Homer1	Synaptic Systems	RRID: AB_10549720
polyclonal chicken anti-GFP	Abcam	RRID: AB_300798
Alexa 647 coupled anti-mouse	ThermoFisher	RRID: AB_2535804
Alexa 568 coupled anti-rabbit	Thermo Fisher Scientific	RRID: AB_10563566
Dylight 405 anti-guinea pig	Jackson Immuno research	RRID: AB_2337432
Monoclonal mouse anti-synaptotagmin-1	Synaptic System	RRID: AB_887832

**Chemicals**		

NaCl	Sigma-Aldrich	7647-14-5
MgCl2	Sigma-Aldrich	7791-18-6
CaCl2	Sigma-Aldrich	10035-04-8
HEPES	Sigma-Aldrich	7365-45-9
d-Glucose	Sigma-Aldrich	50-99-7
Tetrodotoxin	Sigma-Aldrich	554412
Picrotoxin	Sigma-Aldrich	124-87-8
K-gluconate	Sigma-Aldrich	299-27-4
EGTA	Sigma-Aldrich	67-42-5
ATP	Sigma-Aldrich	34369-07-8
GTP	Sigma-Aldrich	56001-37-7

**Experimental models: Organisms/strains**		

Sprague-Dawley rats	Janvier labs	–

### Experimental model and study participant details

#### Hippocampal neuron culture

Sprague-Dawley pregnant rats (Janvier Labs, Saint-Berthevin, France) were sacrificed according to the European Directive rules (2010/63/EU). Dissociated hippocampal neurons from E18 Sprague-Dawley rat embryos of either sex were prepared as described previously (Kaech and Banker, 2006) at a density of 200,000 cells per 60-mm dish on poly-L-lysine pre-coated 1.5H coverslips (Marienfeld, cat. No. 117 580). Neuron cultures were maintained in Neurobasal Plus medium supplemented with 0.5 mM GlutaMAX and 1X B-27 Plus supplement (Thermo Fischer Scientific). Two μM Ara-C were added after 72 hours. Neurons were kept at 37°C and 5% CO2 for 14–16 days.

#### Ethical approval

All experiments were approved by the Regional Ethical Committee on Animal Experiments of Bordeaux. Experiments with wild type rat strictly follow the Directive 2010/63/EU of the European Parliament and of the Council of 22 September 2010 on the protection of animals used for scientific purposes, which corresponds with the French law published on November 12, 2012 in the form of 2 decree (Code Rural R214-87 to 138) and 4 bylaws. Our projects are assessed by the Ethics Committee N° 50 of Bordeaux attached to the CNREEA (Centre national of ethical reflection on animal experiments), and the animal facility is referred under the ethical number B33-063-940.

### Method details

#### Plasmids/transfection

Banker neurons were transfected with WT and T19A mutant of PSD-95, as well as soluble EGFP plasmids using a calcium phosphate protocol (described in^39^).

#### Electrophysiology

mEPSC recordings in neuronal culture were performed as described in.^39^ Briefly, extracellular recording solution was composed of the following (in mM): 110 NaCl, 5 KCl, 2 CaCl_2_, 2 MgCl_2_, 10 HEPES, 10 D-Glucose, 0.0005 Tetrodotoxin, 0.1 Picrotoxin (pH 7.4; ∼256 mOsm/L). The pipettes were filled with intracellular solution composed of the following (in mM): 100 K-gluconate, 10 HEPES, 1.1 EGTA, 3 ATP, 0.3 GTP, 0.1 CaCl_2_, 5 MgCl_2_ (pH 7.3; 230 mOsm). Recordings were performed using an EPC10 patch clamp amplifier operated with Patchmaster software (HEKA Elektronik). Whole-cell voltage clamp recordings were performed at room temperature and at a holding potential of -70mV. Unless specified otherwise, all chemicals were purchased from Sigma-Aldrich except for drugs, which were from Tocris Bioscience.

mEPSC analysis was performed using software developed by Michel Goillandeau, Detection Mini. For the purpose of analysis, events had to exceed a threshold of three times the SD of the baseline noise and only events detected with an amplitude between 5 and 50 pA were taken into account. Series resistance was always lower than 20 MΩ. Similar methods were used for spontaneous EPSCs in neuronal culture. Extracellular recording solution was composed of the following (in mM): 110 NaCl, 5 KCl, 0.2 / 2 / 4 CaCl_2_, 10 HEPES, 10 D-Glucose, 0.1 Picrotoxin (pH 7.4; ∼256 mOsm/L). The pipettes were filled with intracellular solution composed of the following (in mM): 100 K-gluconate, 10 HEPES, 1.1 EGTA, 3 ATP, 0.3 GTP, 0.1 CaCl_2_, 5 MgCl_2_ (pH 7.3; 230 mOsm). The area and duration of individual events were measured using Clampfit 10.7 (Molecular Devices). A template-based search of events was used to obtain the parameters.

#### Labeling

For confocal imaging, primary neuronal cultures were treated either with 30 μM NMDA (Tocris) for 3 minutes or with 100 μM ATP in the presence of CGS15943 (3 μM) (Sigma-Aldrich) for 1 minute and fixed with PFA 30 minutes,3, 6 or 12 hours after, as precised in the figure. PFA was quenched with NH_4_Cl 50 mM for 5 minutes. A permeabilization step with 0.2% triton X100 for 5 minutes was then performed. Cells were washed three times for 5 min in 1x PBS. After three washes with 1x PBS, unspecific staining was blocked by incubating coverslips in 1% BSA for 1h at room temperature. Cells were then incubated with adequate antibodies: for PSD95: monoclonal mouse anti-PSD-95 antibody (MA1-046, ThermoFischer), for Bassoon: monoclonal rabbit anti-Bassoon (141003, Synaptic Systems), for Homer 1, monoclonal Guinea Pig (160004, Synaptic Systems); For GFP, monoclonal chicken anti-GFP (1020, Abcam), all diluted in 1% BSA at 1/500, at room temperature for 4 hours. Coverslips were rinsed three times in 1% BSA solution and incubated in 1% BSA for 1h at room temperature. Primary antibodies were revealed with secondary antibodies: Alexa 647 coupled anti-mouse (A21235, ThermoFisher); Alexa 568 coupled anti-rabbit (A10042, Thermo Fischer Scientific); Alexa 488 coupled anti-chicken (A11039, Thermo Fisher Scientific); Dylight 405 anti-guinea pig (106-475-003 Jackson Immuno research).

#### Confocal imaging

Images were acquired with a Leica TCS SP8 microscope confocal head mounted on a DM6 FS upright stand (Leica Microsystems, Mannheim, Germany), an HC Plan Apo CS2 40X oil NA 1.3 objective and an internal hybrid detector.

Images were acquired on different Z plans and reconstructed as Z projections using ImageJ. Reconstructed images were then analyzed using MetaMorph (Molecular Devices). To measure puncta density, puncta (PSD95 labeling) were automatically thresholded in function of their intensity, then labelled as regions of interest (ROI) on three to five portions of dendrites of around 25 μm length each. ROI surrounding the dendrite are counted, and the dendrite length is measured using the polyline tools in Metamorph. Only dendrites of the first three orders are selected for spine density determination.

#### Live imaging

For live imaging of EGFP-transfected primary neuronal cultures and synaptogamin-1 uptake measurement, neurons were treated with 30 μM NMDA (Tocris) for 3 minutes. After 30 minutes of incubation, they were placed in a Ludin chamber with culture media from their original dish, and a fluorescently labelled monoclonal mouse anti-synaptotagmin-1 (Synaptic System, 105311CpH) was applied in the bath at 1/200 for 30 minutes.

Images were acquired 3 hours after treatment using a Leica DMI8 spinning disk microscope (Leica Microsystems, Wetzlar, Germany) equipped with a confocal Scanner Unit CSU-W1 T2 (Yokogawa Electric Corporation, Tokyo, Japan) with an HCX PL Apo CS2 63X oil NA 1.4 TIRF objective. The system comprised a sCMOS Prime 95B camera (Photometrics, Tucson, USA). The LASER diodes used were at 488 nm (400 mW), and 642 nm (100 mW). Z stacks were done with a galvanometric stage (Leica Microsystems, Wetzlar, Germany). The 37°C and 5 % CO_2_ atmosphere was created with an incubator box and an air heating system (PeCon GmbH, Germany). This system was controlled by MetaMorph software (Molecular Devices, Sunnyvale, USA).

Images were analyzed using MetaMorph. Using the EGFP signal, spines were selected manually as region of interests of a size sufficient to comprise the full spine. All visible spines from a neuron were selected. The distance between spines is estimated by measuring with Metamorph the distance at the dendritic level between the initiation of two protrusions (begining of the spine neck).

To rule out cross-interactions between the parameters of activity and distance from neighboring spines, we selected spines presenting similar parameters between maintained and pruned spines, except the one of interest. To analyze the activity of neighboring spines, maintained spines were selected with activity similar to that of pruned spines (mean pruned spines +/- 2000 a.u.). Only pruned spines with neighbors closer than 5 μm were used. To analyze the distance from surrounding spines, spines with a similar level of activity and surrounded by moderately active synapses (activity of the spine of interest +/- 1000 a.u.) were selected for the maintained group.

### Quantification and statistical analysis

#### Sampling and statistics

Summary statistics are presented as mean ± SEM (Standard Error of the Mean). Each experiment comprised at least three independent repetitions on three different dissections. Both acquisition and analysis were done in blind mode. Statistical significance tests were performed using GraphPad Prism software (San Diego, CA). Normality tests were performed with D’Agostino and Pearson omnibus tests. For non-normally distributed data, we applied the Mann-Whitney test or the Wilcoxon matched-pairs signed rank test for paired observations. When the data followed a normal distribution, we used the paired or unpaired t-test for paired observations unless stated otherwise. The ANOVA test was used to compare means of several groups of normally distributed variables. Indications of significance correspond to p values <0.05(∗), p < 0.005(∗∗), and p<0.0005(∗∗∗). After ANOVA analysis, we applied Dunnett’s post test to determine the p value between two conditions. Results of these tests are noted Anova post-test.
