# Supplementary material for: Synaptic pruning following NMDAR-dependent LTD preferentially affects isolated synapses
Source: iScience. 2025 Aug 29;28(10):113093. doi: 10.1016/j.isci.2025.113093 (PMC12478112; doi:10.1016/j.isci.2025.113093)
Supplement: Document S1. Figures S1 and S2 [file mmc1.pdf]

iScience, Volume *28*

Supplemental information

Synaptic pruning following NMDAR-dependent LTD preferentially affects isolated synapses

Côme Camus, Léa Leval, Viviana Villicana-Munoz, Sarka Jelinkova, Benjamin Compans, Frédéric Gambino, Etienne Herzog, Daniel Choquet, and Eric Hosy

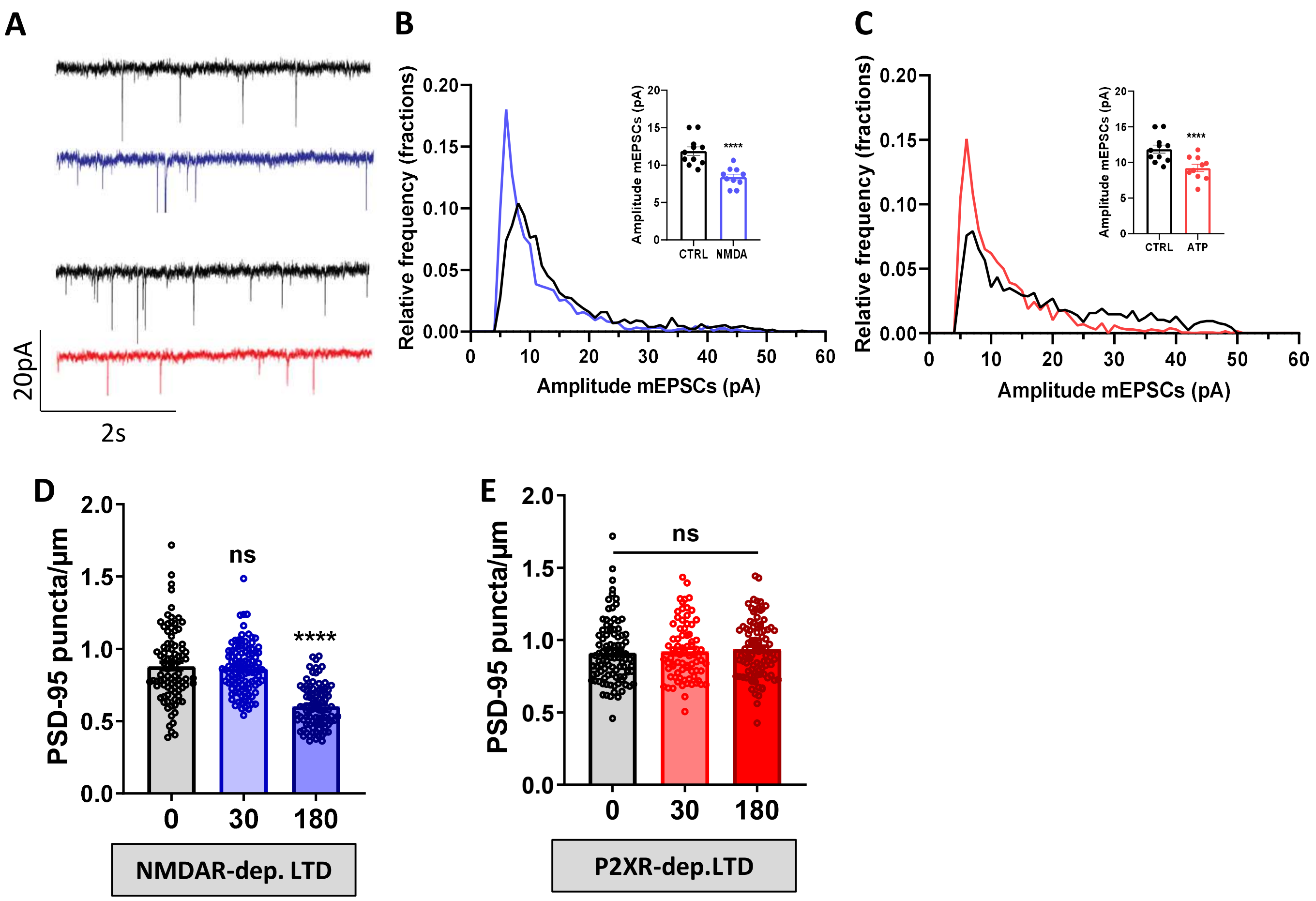

Figure 1 : NMDAR-dependent but not ATP-dependent LTD induces synaptic pruning. A: Example of traces of mEPSCs recordings when LTD is induced with either NMDA or ATP treatment. Control in black, NMDA + 30 min in blue and ATP + 30 min in red. B and C: Left, frequency distribution of mEPSCs amplitude before and 30 min following LTD induction with NMDA (B) and ATP (C). Right: Median of mEPSCs amplitudes, one dot representing a median of a cell. (mean +/- SEM, t-test, p = 0.0001 for B, p<0.0001 for C). D and E: quantification of PSD-95 puncta density before (black), 30 min (light color) and 3 hours (dark color) following LTD induction with NMDA (E, blue) or ATP (F, red). Mean +/- SEM, one dot represents one portion of dendrite (one-way ANOVA).

Supplementary Figure 1.1

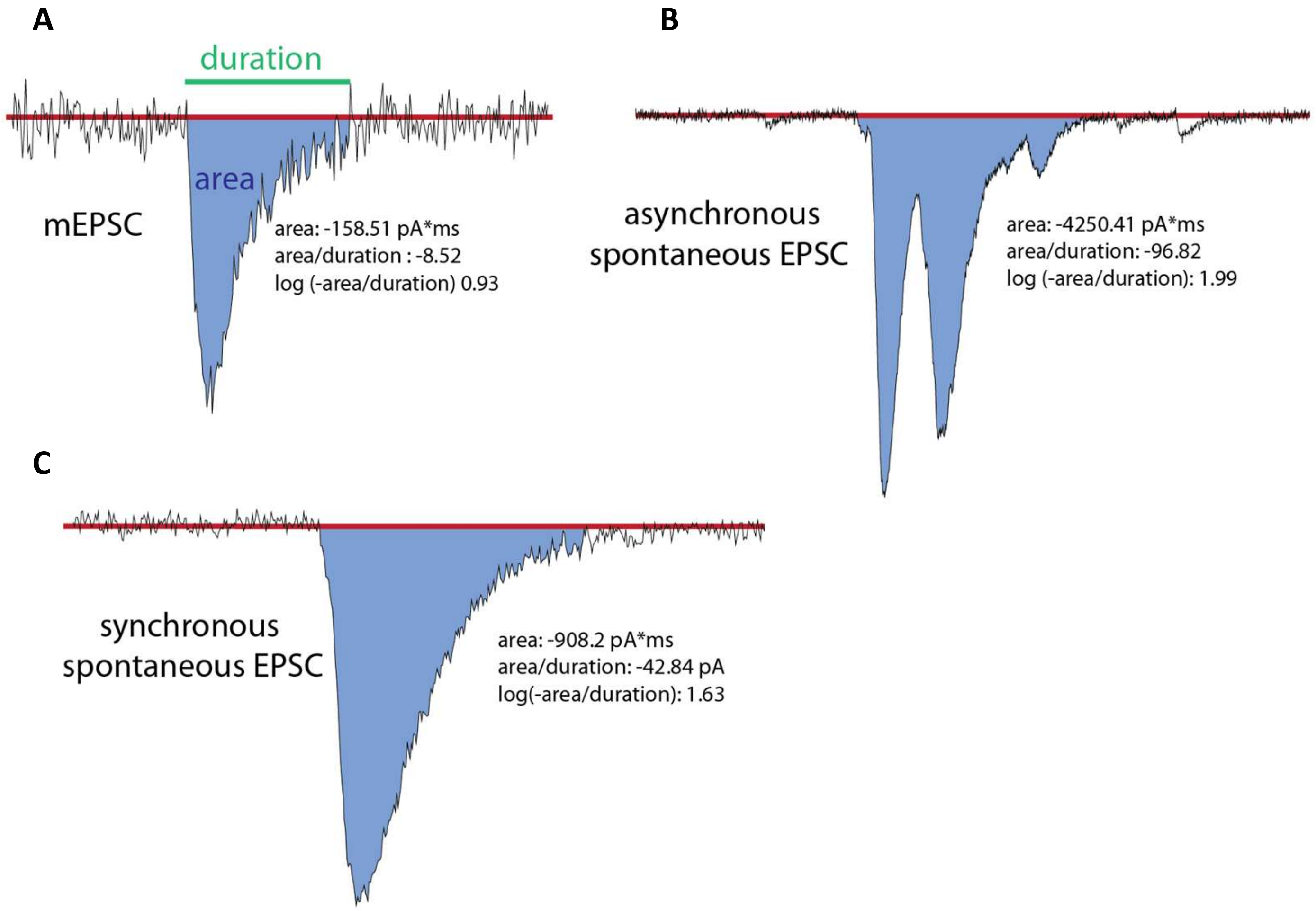

Supplementary figure 6.1: example traces of the different types of recorded EPSCs. A. Example trace of a mEPSC. The area used for analysis correspond to the blue part, and is determined as the surface between the baseline (in red) and the line of current of the event. The duration (green line) is the length of the mEPSC. B: Example trace of an asynchronous spontaneous EPSC, and C: example trace of a synchronous spontaneous EPSC. Data on the right of each trace correspond to the extracted parameters of the shown event.

Supplementary Figure 6.1

The different types of synaptic events
